# Supplementary material for: Multiomics analysis of GSTP1 knockdown pancreatic cancer cells reveals key regulators of redox and metabolic homeostasis
Source: Biol Open. 2025 Aug 14;14(8):bio061986. doi: 10.1242/bio.061986 (PMC12381923; doi:10.1242/bio.061986)
Supplement: Supplementary information [file biolopen-14-061986-s1.pdf]

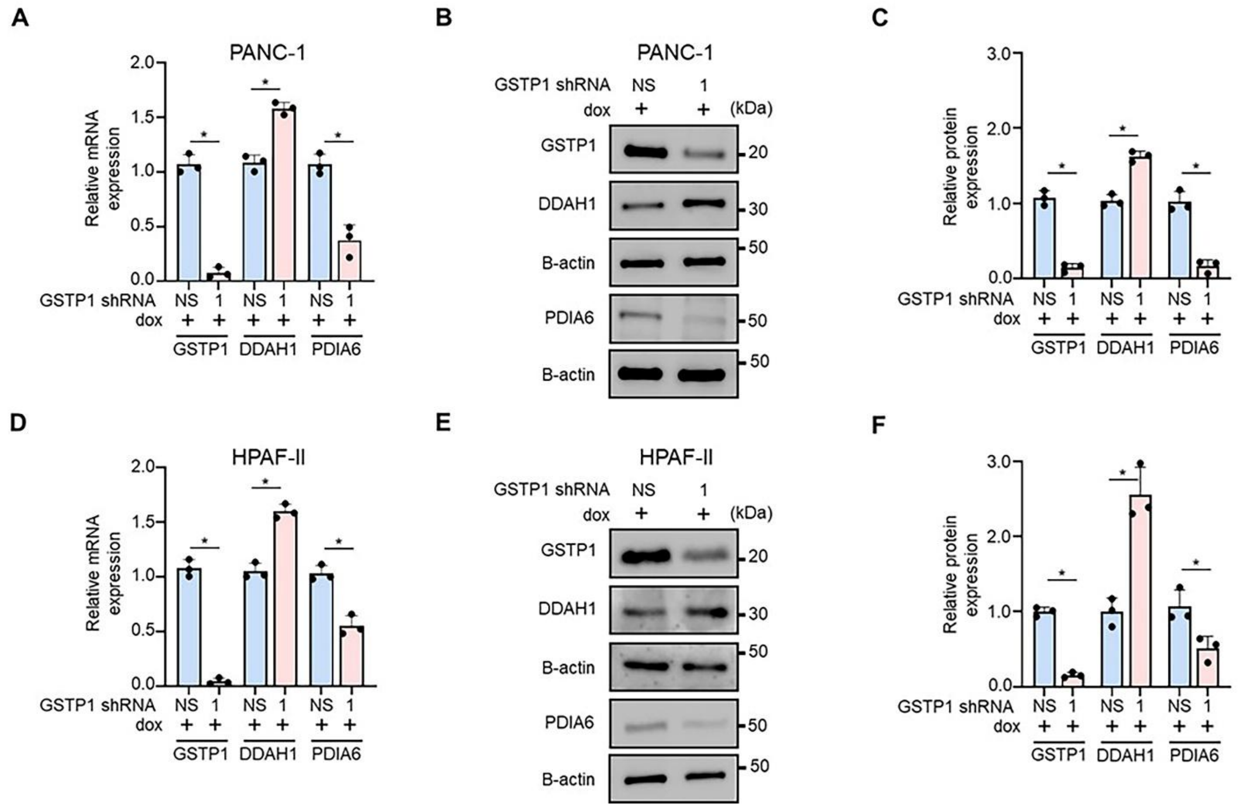

Fig. S1. Gene regulation following GSTP1 knockdown in PANC-1 and HPAF-II cells

(A) qRT-PCR analysis of PDIA6 and DDAH1 mRNA expression in PANC-1 cells with GSTP1 knockdown (shGSTP1-1). (B, C) Western blot validation of PDIA6 and DDAH1 protein levels. (D-F) Similar experiments were performed in HPAF-II cells, where mRNA and protein levels were quantified following GSTP1 knockdown. Results were compared between NS control cells and shGSTP1-1 knockdown. All data represent three independent experiments (n = 3). A Student's t-test was used to determine statistical significance across groups. * denotes significant differences between knockdown and control groups ($p < 0.05$). Error bars represent standard deviation.

Biology Open • Supplementary information

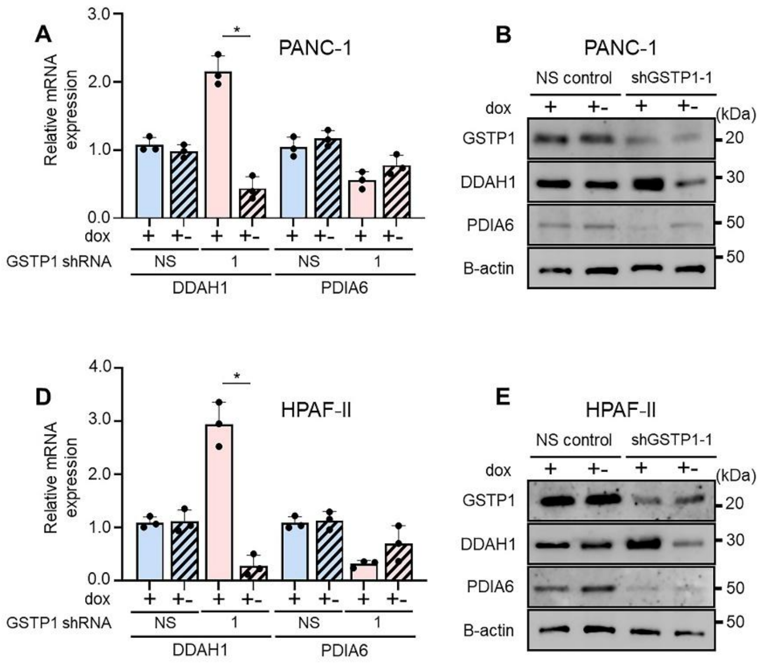

Fig. S2. Recovery of GSTP1 knockdown in PANC-1 and HPAF-II cells (A) After 120 hours of GSTP1 restoration (dox +-), qRT-PCR measured PDIA6 and DDAH1 expression in PANC-1 cells. (B, C) Western blot quantification of protein expression. (D-E) Corresponding mRNA and protein measurements were taken in HPAF-II cells following GSTP1 knockdown and recovery. NS control samples were compared with shGSTP1-1. Images are representative of three independent replicates (n = 3). Statistical significance between conditions was evaluated using Student's t-test, with * indicating $p < 0.05$. Error bars represent standard deviation.

Biology Open • Supplementary information

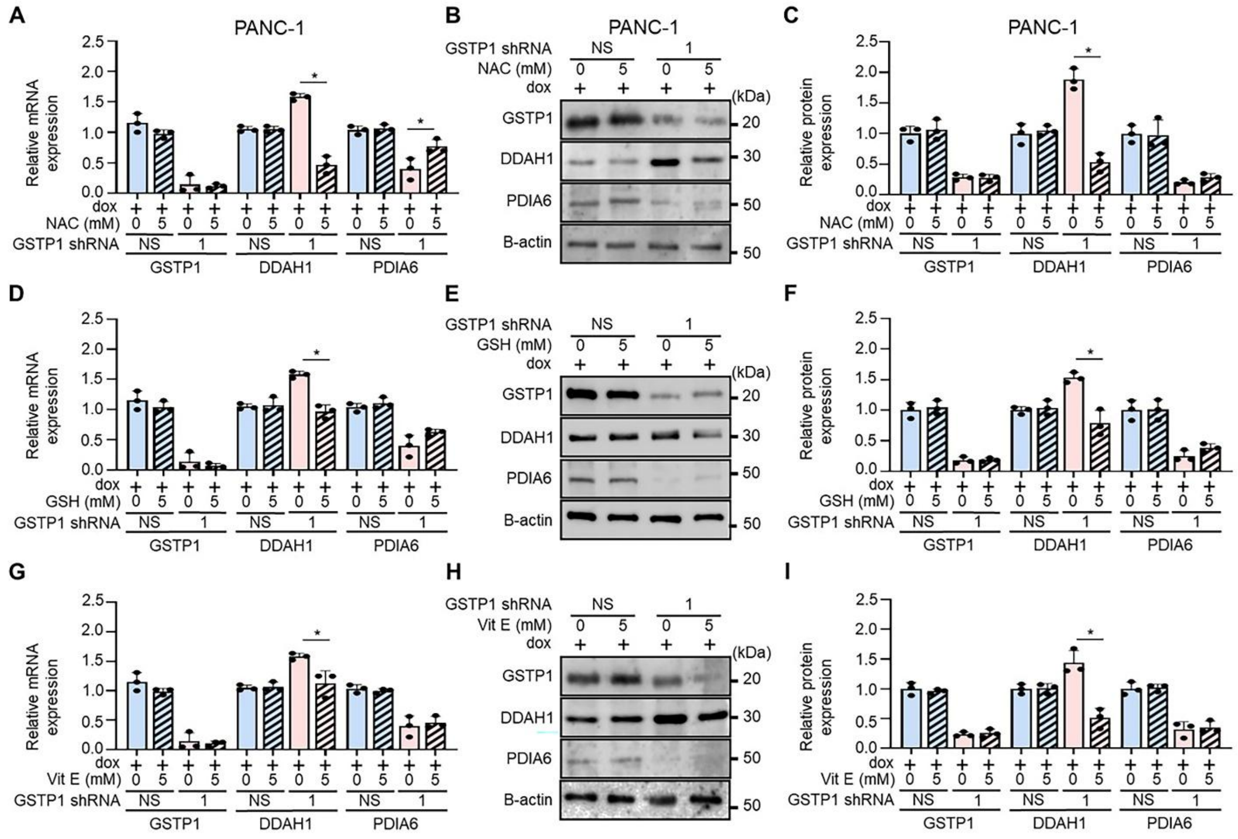

Fig. S3. Redox-dependent expression regulation in GSTP1 knockdown PANC-1 cells. The impact of redox-modulating agents on RNA and protein levels of GSTP1, PDIA6, and DDAH1 was examined in PANC-1 knockdown cells. (A) qPCR results following 48-hour N-acetyl cysteine (NAC) treatment in GSTP1 knockdown cells. (B, C) Western blot validation of NAC effects on protein levels. (D-F) Expression analysis following glutathione (GSH) treatment. (G-I) Expression analysis following Vitamin E (Vit. E) treatment. Data from NS control cells were compared with shGSTP1-1. Each result reflects three independent experiments (n = 3). Student's t-test determined significance, with * marking $p < 0.05$. Error bars represent standard deviation.

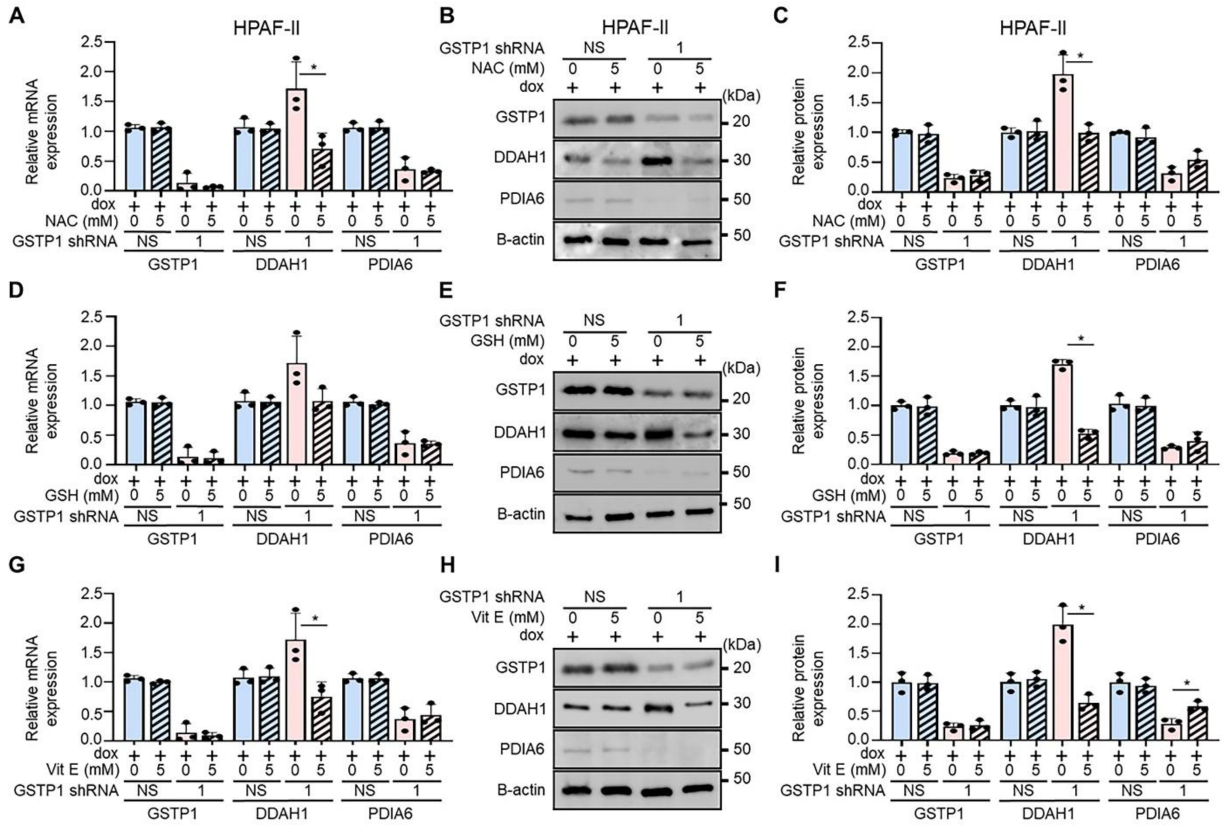

Fig. S4. Redox-dependent regulation of gene expression in GSTP1 knockdown HPAF-II cells. The expression of GSTP1, PDIA6, and DDAH1 in GSTP1 knockdown HPAF-II cells was measured after redox treatments. (A) qPCR results following 48 hour N-acetyl cysteine (NAC) treatment. (B, C) Western blot validation protein levels. (D-F) Expression analysis following glutathione (GSH) treatment. (G-I) Expression analysis following Vitamin E (Vit. E) treatment. Data were compared between NS control and shGSTP1-1 knockdown samples. All results represent three independent experiments (n = 3). Statistical analysis was conducted using Student's t-test, with * indicating $p < 0.05$. Error bars represent standard deviation.

Biology Open • Supplementary information

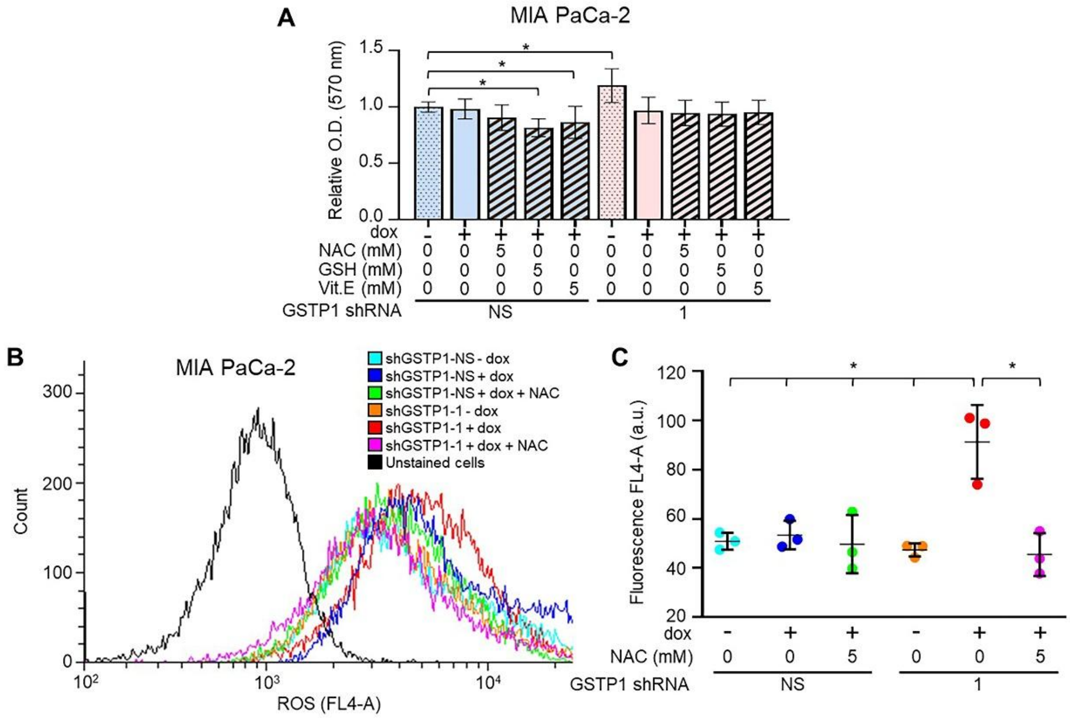

Fig. S5. Antioxidant treatment does not significantly alter viability but restores ROS levels to baseline in GSTP1 knockdown cells. (A) MTT assay results following 96 hours of doxycycline treatment and subsequent 48-hour antioxidant treatment in MIA PaCa-2 cells. GSTP1 knockdown (shGSTP1-1) and non-specific control (NS) cells were treated with N-acetylcysteine (NAC, 5 mM), glutathione (GSH, 5 mM), or Vitamin E (Vit.E, 5 mM). Statistical comparisons were made relative to the NS -doxy control group. (B) Representative flow cytometry histogram showing ROS levels, detected by CellROX™ DeepRed fluorescence (FL4-A), in NS and shGSTP1-1 cells treated with doxycycline and/or NAC as indicated. (C) Quantification of FL4-A fluorescence intensities shows a significant increase in ROS with GSTP1 knockdown (shGSTP1-1 + doxy), which is restored to baseline levels by NAC treatment. Data are representative of three independent experiments (n = 3), and statistical significance was evaluated using a Student's t-test (*p < 0.05). Error bars represent standard deviation.

Biology Open • Supplementary information

Table S1. Non-specific control (NS) and GSTP1 knockdown (shGSTP1-1) MIA PaCa-2 cells were sequenced using an Illumina NovaSeq6000 with four replicates each (n=4). The characteristics of the raw output reads were summarized. QC: quality control.

Sample	QC Passed reads	% overall alignment	% unique alignments	% successfully assigned alignments
MIA PaCa2 NS (Rep 1)	25,776,715	97.07	89.65	71.8
MIA PaCa2 NS (Rep 2)	32,194,825	97.05	89.81	73.1
MIA PaCa2 NS (Rep 3)	25,783,621	97.02	89.69	72.7
MIA PaCa2 NS (Rep 4)	27,552,146	96.84	89.33	72.6
MIA PaCa2 shGSTP1-1 (Rep 1)	32,942,440	96.68	89.93	74.3
MIA PaCa2 shGSTP1-1 (Rep 2)	21,998,409	96.96	89.96	74.0
MIA PaCa2 shGSTP1-1 (Rep 3)	21,904,880	96.81	90.02	74.5
MIA PaCa2 shGSTP1-1 (Rep 4)	21,162,205	96.73	89.81	74.6

Table S2. Primer sequences used for measuring mRNA expression via quantitative polymerase chain reaction.

Gene	Forward primer	Reverse primer
GSTP1	5′-CAG GAG GGC TCA CTC AAA GC-3′	5′-AGG TGA CGC AGG ATG GTA TTG-3′
PDIA6	5′-GGT GTT TGT GAG GCG TAG AT -3′	5′- CCA TCA GCA GTG GAA GGA TAA A-3′
DDAH1	5′-AAC TCA CTG TGC CTG ATG AC-3′	5’-ACC TTT GCA CTT TCT GGA TAC T-3’
β-Actin	5′-TTG CCG ACA GGA TGC AGA A-3′	5′-GCC GAT CCA CAC GGA GTA CTT-3′
β-Tubulin	5′-GTT CGC TCA GGT CCT TTT GG-3′	5′-CCC TCT GTG TAG TGG CCT TTG-3′

Biology Open • Supplementary information
